# Nitrate-Induced CLE Peptide Systemically Inhibits Nodulation in *Medicago truncatula*

**DOI:** 10.3390/plants9111456

**Published:** 2020-10-28

**Authors:** Maria Lebedeva, Mahboobeh Azarakhsh, Yaroslavna Yashenkova, Lyudmila Lutova

**Affiliations:** 1 Department of Genetics and Biotechnology, Saint Petersburg State University, Universitetskaya emb.7/9, 199034 Saint Petersburg, Russia; mahboobeazarakhsh@kub.ac.ir (M.A.); st050678@student.spbu.ru (Y.Y.); l.lutova@spbu.ru (L.L.); 2 Cell and Molecular Biology Department, Kosar University of Bojnord, Bojnord 9415615458, Iran

**Keywords:** symbiotic nodules, autoregulation of nodulation, nitrate, CLE, CLV1-like kinase

## Abstract

Legume plants form nitrogen-fixing nodules in symbiosis with soil bacteria rhizobia. The number of symbiotic nodules is controlled at the whole-plant level with autoregulation of nodulation (AON), which includes a shoot-acting CLV1-like receptor kinase and mobile CLE (CLAVATA3/ENDOSPERM SURROUNDING REGION-related) peptides that are produced in the root in response to rhizobia inoculation. In addition to rhizobia-induced CLE peptides, nitrate-induced *CLE* genes have been identified in *Lotus japonicus* and *Glycine max*, which inhibited nodulation when overexpressed. However, nitrate-induced *CLE* genes that systemically suppress nodulation in AON-dependent manner have not been identified in *Medicago truncatula*. Here, we found that *MtCLE35* expression is activated by both rhizobia inoculation and nitrate treatment in *M. truncatula*, similarly to *L. japonicus CLE* genes. Moreover, we found that MtCLE35 systemically suppresses nodulation in AON-dependent manner, suggesting that MtCLE35 may mediate nitrate-induced inhibition of nodulation in *M. truncatula*.

## 1. Introduction

The *Rhizobium*–legume interaction results in the formation of new organs on plant roots—symbiotic nodules, where the process of nitrogen fixation takes place. Nodule number is regulated by the plant systemically, and this process is known as autoregulation of nodulation (AON) [1]. A key component of AON is a CLAVATA 1 (CLV1)-like receptor kinase encoded by *SUPER NUMERIC NODULES (MtSUNN)* in *Medicago truncatula, HYPER NODULATION ABERRANT ROOT FORMATION1* (*LjHAR1*) in *Lotus japonicus,* and *NODULE AUTOREGULATION RECEPTOR KINASE* (*GmNARK*) in *Glycine max* [2,3,4]. Legume mutants carrying mutations in these genes exhibit supernodulating phenotype (they form an excessive number of nodules), and some of them are nitrate tolerant (nodulation is not suppressed by high nitrogen concentrations which inhibit nodulation in wild-type plants) [5,6,7]. A grafting experiment demonstrated that the supernodulating phenotype of mutants defective in CLV1-like receptor kinase is determined by the shoot part of the plant [8]. Therefore, a signaling cascade activated by CLV1-like kinase in the shoot suppresses the subsequent nodulation on the roots systemically through a shoot-derived inhibitor, thereby regulating nodule number [1,9,10,11]. CLE (CLAVATA3/ENDOSPERM SURROUNDING REGION-related) peptides represent mobile signal molecules that are produced in the root in response to rhizobia inoculation and trigger AON through shoot-acting CLV1-like receptor kinase [12,13,14]. For *L. japonicus* CLE peptide, LjCLE-RS2, produced in the root in response to rhizobia inoculation, the presence in xylem sap collected from the shoot and the direct binding to LjHAR1 CLV1-like receptor kinase were shown [15].

In response to AON activation, the expression of cytokinin biosynthesis gene *LjIPT3* was increased in the shoot in LjHAR1-dependent manner, and shoot-to-root transported cytokinin was suggested to inhibit nodule initiation in *L. japonicus* [16]. For the *MtIPT3* gene in *M. truncatula* AON-dependent activation in the shoot was also shown [17]; however, the activation of orthologous gene in soybean, *GmIPT5,* appeared to be AON-independent [18]. Moreover, the inhibition of shoot-to-root auxin transport was shown to occur downstream of AON activation [19]. Finally, in response to AON the production of miR2111 is reduced in the shoot [10,11]. miR2111 is believed to be a mobile microRNA that is transported from the shoot to the root and increases the competence of the root to rhizobia infection and nodulation via down-regulation of the *TML* gene, which encodes F-box protein—a negative regulator of symbiosis [10,20]. Overproduction of miR2111 results in hyperinfection and increased nodule number [10]. AON inhibits miR2111 synthesis in the shoot, thereby limiting nodule number produced on the inoculated roots [10,11].

CLE peptides inhibiting nodulation have been identified in different legumes: MtCLE13 and MtCLE12 in *M. truncatula* [12], LjCLE-RS1, LjCLE-RS2, and LjCLE-RS3 (CLE-ROOT SIGNAL) in *L. japonicus* [13,21], GmRIC1 and GmRIC2 (RHIZOBIUM INDUCED CLE) in *G. max* [14]. The expression of these genes is activated in response to rhizobia inoculation, and their overexpression inhibited nodulation in AON-dependent manner [12,13,14]. Nitrate is a well-known regulator of nodulation, since nodule formation takes place at low nitrate level in the soil, whereas high nitrate doses inhibit nodulation [22]. In soybeans and *L. japonicus*, in addition to rhizobia-induced CLE peptides, nitrate-induced *CLE* genes have been identified, which are supposed to mediate nitrate-dependent suppression of symbiotic nodule development [13,14,23].

In *L. japonicus*, the expression of *LjCLE-RS2* and *LjCLE-RS3* genes is activated by both rhizobia and nitrate treatment [13,21]. However, in soybean in addition to rhizobia-induced CLE peptides that systemically suppress nodulation, nitrate-induced CLE peptides (NIC), GmNIC1 and GmNIC2, have been identified, that are activated in response to nitrate treatment and inhibit nodulation locally in the root [14,23]. GmNIC1 and GmNIC2 overexpression in transgenic roots obtained by *Agrobacterium rhizogenes*-mediated transformation inhibited nodulation locally, but did not suppress nodulation on non-transgenic roots, which indicates the absence of systemic effect on nodulation [14]. Therefore, in contrast to *L. japonicus CLE* genes, which are activated by both rhizobia and nitrate and act systemically to suppress nodulation, in soybean there are two groups of CLE-peptides: RIC are induced by rhizobia and systemically suppress nodulation, whereas NIC are activated in response to nitrate treatment and inhibit nodulation locally in the root via root-acting CLE receptor. The mechanism underlying the specific perception of CLE peptide by a shoot and a root-acting receptor are of great interest and remain to be elucidated.

In *M. truncatula*, two CLE-peptides, MtCLE12 and MtCLE13, were found to be involved in AON [12]. The expression of these two genes was induced in response to rhizobia inoculation, whereas their induction by nitrate has not been shown [12]. Ectopic expression of these genes systemically suppressed nodulation in wild-type plant, but not in MtSUNN mutant lacking a functional CLV1-like receptor kinase, indicating that MtSUNN may be responsible for MtCLE12 and MtCLE13 perception in the shoot. Recently, an additional close homologue of *MtCLE12* and *MtCLE13* has been identified in *M. truncatula* genome—the *MtCLE35* gene [24,25]. The expression of this gene is induced in rhizobia according to transcriptomic data [25]. However, the role of the *MtCLE35* gene in AON has not been investigated. Here, we found that *MtCLE35* expression is activated in response to rhizobia inoculation as well as to nitrate treatment, similarly to *L. japonicus CLE* genes. Moreover, we found that MtCLE35 systemically suppresses nodulation in an AON-dependent manner, suggesting that MtCLE35 is involved in nitrate-induced inhibition of nodulation in *M. truncatula*.

## 2. Results

### 2.1. MtCLE35 Is Expressed during Nodulation and in Response to Nitrate Treatment

**Figure 1:**
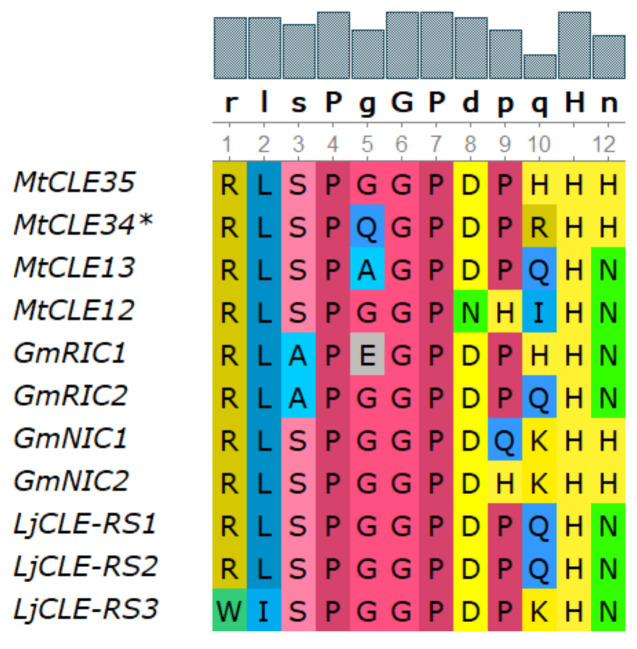
Sequence alignment of CLAVATA3/ENDOSPERM SURROUNDING REGION-related (CLE) domains from *Medicago truncatula, Glycine max and Lotus japonicus* nodulation-suppressing CLE peptides.

According to phylogenetic analysis, the *MtCLE35* gene (Medtr2g091125.1) is closely related to other genes encoding nodulation-suppressing CLE peptides [24] (see Figure S1). The amino acid sequence of CLE domain of MtCLE35 shares high similarity with CLE domain sequences of other nodulation-suppressing CLE peptides, and differs from that of GmNIC1,2 and LjCLE-RS1,2 only at two positions (Figure 1). Moreover, MtCLE35 contains the consensus sequence TLQAR in the signal peptide domain, which was previously found in other nodulation-suppressing CLE peptides [24,26].

**Figure 2:**
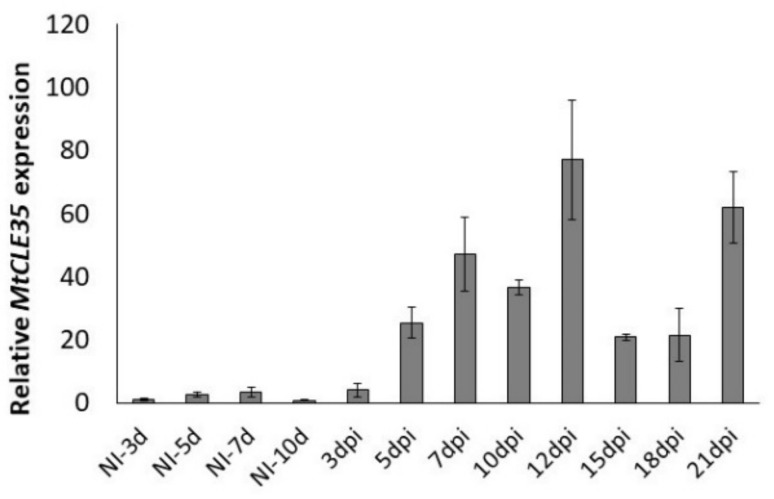
The expression levels of the *MtCLE35* gene at different days post inoculation (dpi) in comparison to the non-inoculated roots (NI). Results are mean ± standard error of the mean (SEM) of three technical repeats of one biological repeat, representative for three independent experiments.

To study the expression dynamics of the *MtCLE35* gene during nodule development, the relative transcript levels of *MtCLE35* were analyzed at different stages after inoculation using quantitative reverse transcription polymerase chain reaction (qRT-PCR) (from 3 to 21 dpi (days post inoculation)) in comparison to the non-inoculated roots (NI). The induction of *MtCLE35* expression was observed at 5 dpi. At later stages of nodulation its expression level increased reaching peak at 10–12 dpi, and after a slight reduction at 15–18 dpi, it was increased again in mature nodules at 21 dpi (Figure 2). This suggests that *MtCLE35* acts not only at early stages of nodule development but also at later stages in mature nodules. Similar dynamics of *MtCLE35* expression were observed in three independent experiments (see Figure S2 for the data from additional biological repeat).

The activation of *MtCLE35* expression in developing nodules is consistent with transcriptomic data obtained by LCM (Laser Capture Microdissection)-RNA-seq (https://iant.toulouse.inra.fr/symbimics/) for *M. truncatula* [27] (Figure S3A). Moreover, *MtCLE35* expression was also increased in developing nodules according to Small Secreted Peptide Gene Expression Atlas (SSP-GEA) available in the *Medicago truncatula* Small Secreted Peptide Database (https://mtsspdb.noble.org/ [28]) (Figure S3B).

**Figure 3:**
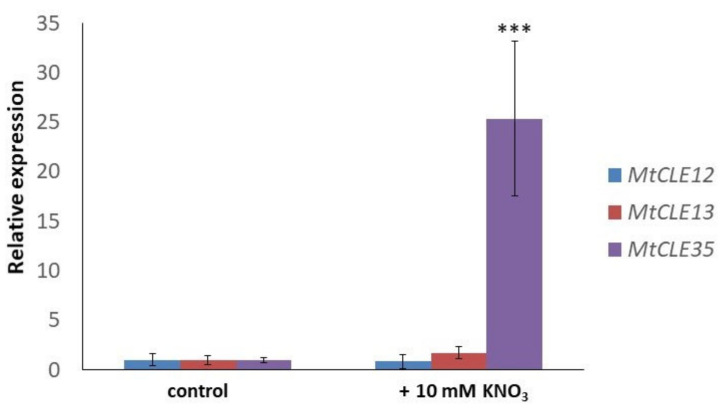
The expression levels of *MtCLE12, MtCLE13,* and *MtCLE35* in response to nitrate treatment (10 mM KNO_3_). Asterisks indicate statistically significant differences in *MtCLE35* expression compared with control (*p* < 0.001).

Next, we checked the effect of nitrate treatment on the *MtCLE35* gene expression. After 24 h of nitrate treatment (10 mM KNO_3_), the expression level of the *MtCLE35* gene was 25-fold increased (Figure 3). However, no increase of *MtCLE12* and *MtCLE13* in response to nitrate has been revealed. This suggests that *MtCLE35* is a nitrate-responsive gene.

### 2.2. Overexpression of MtCLE35 Suppresses Nodulation in Wild-Type Plants

**Figure 4:**
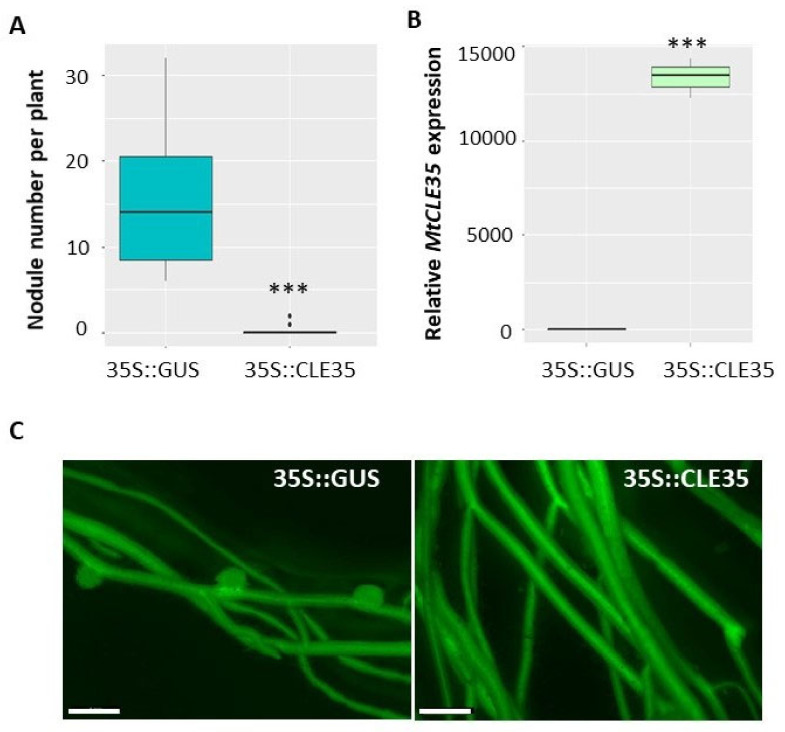
*MtCLE35* overexpression inhibits nodulation in wild-type plants. (**A**) Nodule number in wild-type (A17) A green fluorescent protein (GFP)-positive transgenic roots transformed with the *35S*::*GUS* (control) or *35S*::*MtCLE35* construct. Asterisks indicate statistically significant differences (Mann-Whitney *U* test, *p* < 0.001). (**B**) The expression level of the *MtCLE35* gene in GFP-positive transgenic roots transformed with the *35S*::*GUS* (GUS_OE, control) or *35S*::*MtCLE35* construct. Asterisks indicate statistically significant differences *p* < 0.001. Box plots are presented with median values. (**C**) Examples of the nodulation phenotype of GFP-positive transgenic roots carrying the *35S::GUS* (control) or *35S::MtCLE35* construct on wild-type plants. Bars = 1000 µm.

To explore the role of the *MtCLE35* gene in nodulation we have overexpressed this gene under 35S promoter in *M. truncatula* roots (35S::MtCLE35). *β*-glucuronidase (GUS)-overexpressing roots were used as a control (35S::GUS). green fluorescent protein (GFP) fluorescence was used as a positive marker to select transgenic roots, where *MtCLE35* overexpression was confirmed by qPCR analysis (Figure 4). Nodules were counted on transgenic roots at 21 dpi. In wild type A17 line, overexpression of *MtCLE35* resulted in a significant reduction of nodulation. In the control GUS-overexpressing roots, on average 16 nodules were counted per transgenic root, whereas in *MtCLE35*-overexpressing roots in most cases nodulation was completely inhibited, and only two out of 15 plants carried up to two nodules per transgenic root in each biological experiment (Figure 4).

### 2.3. The Inhibitory Effect of MtCLE35 Overexpression on Nodulation Is Systemic

**Figure 5:**
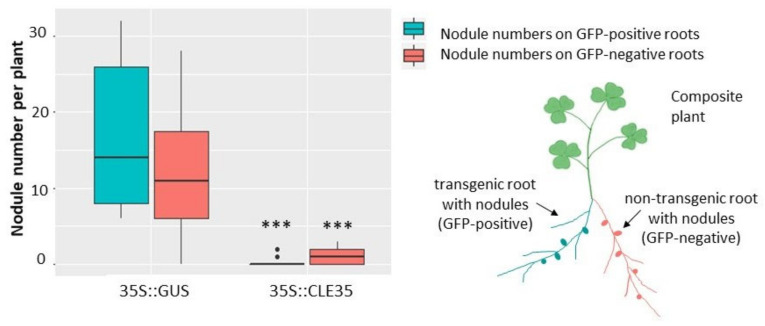
*MtCLE35* overexpression systemically inhibits nodulation in wild-type plants. Nodule number in wild-type (A17) composite plants on green fluorescent protein (GFP)-positive transgenic roots (cyan) carrying the *35S*::*GUS* (control) or *35S*::*MtCLE35* construct and on GFP-negative non-transgenic roots (coral) are represented. Box plots are presented with median values. Asterisks indicate statistically significant differences (Mann-Whitney *U* test, *p* < 0.001).

To check if such inhibitory effect of *MtCLE35* overexpression on nodulation is systemic, we counted nodule numbers not only on transgenic roots, overexpressing *MtCLE35*, but also on non-transgenic root which emerged together with transgenic roots in composite plants after *A. rhizogenes*-mediated transformation. In addition to *MtCLE35* coding DNA sequence) under 35S promoter, the genetic construct used for plant transformation also contains GFP cassette (enhanced green fluorescent protein (eGFP)) under rolD promoter) to select transgenic roots. In this system, GFP-positive roots represent transgenic roots, which overexpress *MtCLE35* (or *GUS* in control plants), whereas GFP-negative roots (that do not demonstrate GFP fluorescence) represent non-transgenic ones (Figure 5). A significant reduction of nodule number was found in both transgenic (GFP-positive, *MtCLE35*-overexpressing) and GFP-negative non-transgenic roots which do not have the *35S::MtCLE35* insert (Figure 5). Representative images of composite wild-type plants containing both transgenic *MtCLE35*-overexpressing roots and non-transgenic roots are presented inFigure S4. Only very few nodules were found on non-transgenic roots in composite plants containing *MtCLE35*-overexpressing roots. This fact suggests a systemic nature of *MtCLE35* inhibitory effect, in which a long-distance transport of *MtCLE35* gene product seems to be involved.

### 2.4. The Effect of MtCLE35 on Nodulation Depends on MtSUNN Receptor Kinase

**Figure 6:**
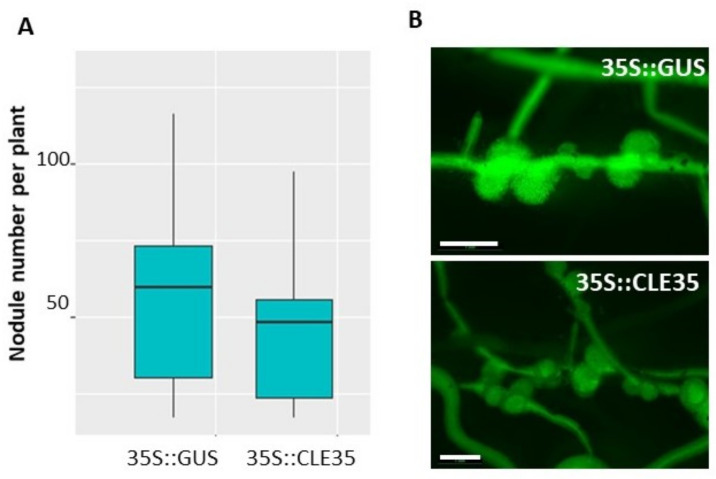
*MtCLE35* overexpression does not affect nodulation phenotype in *sunn-4* mutant. (**A**) Nodule number on green fluorescent protein (GFP)-positive transgenic roots of *sunn-4* plants transformed with the *35S*:*GUS* (control) or *35S*::*MtCLE35* construct. Box plots are presented with median values. No statistically significant differences were found between nodule number in *35S*:*MtCLE35* and *35S*:*GUS* (control) overexpressing plants. (**B**) Examples of the nodulation phenotype of GFP-positive transgenic roots carrying the *35S*::*GUS* (control) or *35S::MtCLE35* construct on *sunn-4* mutant plants. Bars = 1000 µm.

Next, to check if *MtCLE35* action on nodulation depends on MtSUNN receptor kinase, we analyzed the effect of *MtCLE35* overexpression on nodulation in *sunn-4* mutant plants. In contrast to the wild type, *MtCLE35* overexpression had no obvious effect on nodulation in *sunn-4* mutant (Figure 6, Figure S5). Both GUS-overexpressing control roots and *MtCLE35*-overexpressing roots of *sunn-4* mutant plants demonstrated a supernodulating phenotype. No statistically significant difference in nodule numbers was found between control (GUS-overexpressing roots) and *MtCLE35*-overexpressing roots, indicating that the inhibitory effect of *MtCLE35* overexpression on nodulation was abolished in *sunn-4* mutant (Figure 6).

Therefore, functional *MtSUNN* gene is required for *MtCLE35*-dependent inhibition of nodulation in *M. truncatula*.

## 3. Discussion

**Figure 7:**
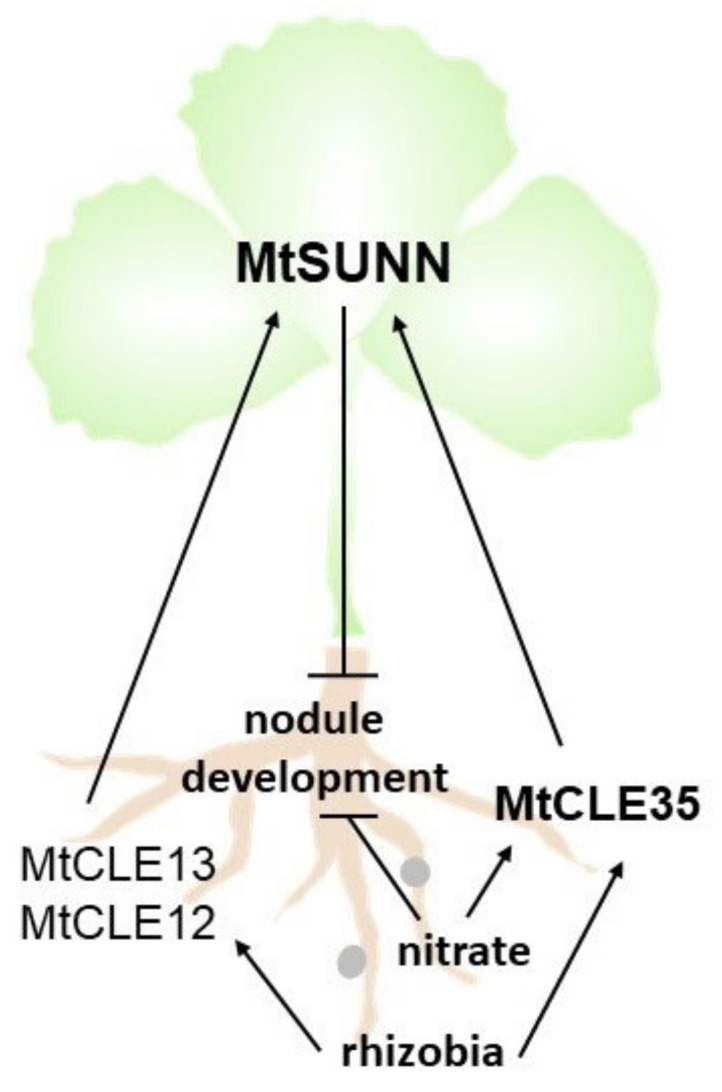
Model of MtCLE35 action in autoregulation of nodulation (AON). The *MtCLE12* and *MtCLE13* genes are induced in response to rhizobia inoculation, whereas the expression of the *MtCLE35* gene is induced by both rhizobia inoculation and nitrate treatment. The CLAVATA3/ENDOSPERM SURROUNDING REGION-related (CLE) peptides encoded by the *MtCLE12, MtCLE13,* and *MtCLE35* genes systemically inhibit nodulation via shoot-acting MtSUNN receptor kinase. MtCLE35 mediates nitrate-dependent inhibition of nodulation.

Here, we found that *MtCLE35* expression is induced both by rhizobia and by nitrate treatment. In contrast, the *MtCLE12* and *MtCLE13* genes that were previously found to be AON-dependent inhibitors of nodulation induced by rhizobia [12] did not exhibit nitrate responsiveness. Moreover, we showed that *MtCLE35* overexpression in transgenic roots inhibited nodulation in wild-type plants, both in transgenic roots and in non-transgenic roots of composite plants bearing *MtCLE35*-overexpressing roots, indicating that MtCLE35 suppresses nodulation systemically. Our results indicate that MtCLE35 systemically inhibits nodulation in an AON-dependent manner, since *MtCLE35* inhibitory effect on nodulation was observed in wild-type plants, but not in *sunn-4* supernodulating mutant defective in AON. Therefore, MtCLE35 peptide is involved in AON, and MtSUNN receptor kinase may act as its receptor in the shoot. Collectively, our data suggest that MtCLE35 is a mediator of nitrate-dependent inhibition of nodulation in *M. truncatula* (Figure 7).

The nitrate-induced *MtCLE35* expression was also described in a recent preprint paper available at bioRxiv [29]. In this paper, the inhibitory effect of MtCLE35 on nodulation was also reported, although it was less pronounced according to the presented data (on average, approximately 1.5 nodules were formed on *MtCLE35*-overexpressing plants compared to approximately three nodules formed in the control plants), and the systemic AON-dependent MtCLE35 action was not investigated in this study.

Nitrate-induced CLE peptides that suppress nodulation when overexpressed have been previously characterized in *L. japonicus* and *G. max*. In *L. japonicus* two CLE peptides, LjCLE-RS2 and LjCLE-RS3, are induced by both nitrate treatment and rhizobia inoculation [13,21], whereas in soybean there are two groups of *CLE* genes induced by different factors: rhizobia-induced *CLE* (*RIC*) and nitrate-induced *CLE* (*NIC*) [14,23]. The *MtCLE35* regulation seems to be similar to that of *L. japonicus* LjCLE-RS2 and LjCLE-RS3, and this could be due to the similarity of their regulatory sequences that can be activated by both nitrate- and nodulation-responsive factors. In soybean, two distinct groups of CLE peptide have evolved: RIC peptides that inhibit nodulation systemically in AON-dependent way, and NIC peptide that act locally in the root to suppress nodulation in response to nitrate. Therefore, in legume plants multiple *CLE* genes have been evolved to control nodulation. Some of them are induced by rhizobia-activated signaling cascade alone, other *CLE* genes are regulated by both rhizobia and nitrate signaling, whereas the other group of *CLE* genes are only nitrate-responsive. A detailed comparative analysis of the regulatory sequences of these *CLE* genes should help to elucidate reasons for such differences in their regulation.

All CLE peptides suppressing nodulation in legumes were shown to act through CLV1-like kinase, and for LjCLE-RS2 the direct binding with LjHAR1 was confirmed [15]. Moreover, recently it was found that MtCLE33 and MtCLE53 peptides are activated by phosphate and arbuscular mycorrhizal fungi, respectively, and regulate mycorrhization in MtSUNN-dependent manner [30]. Therefore, MtSUNN/LjHAR1 should be responsible for the reception of multiple CLE peptides, activating by diverse factors—microsymbionts and nutrients. It is of great interest to study if there are additional receptor proteins that could help MtSUNN/LjHAR1 to distinguish between different CLE peptides and to elucidate how the specific action of the downstream signaling pathway induced by MtSUNN/LjHAR1 is achieved.

## 4. Materials and Methods

### 4.1. Plant Material, Bacterial Strains, and Growth Conditions

*M. truncatula* A17 and *sunn-4* seeds were sterilized with sulfuric acid and washed several times with sterile distilled water, then transferred to the plates with 1% agar for germination. Plates were held at +4 °C for one day, then seeds were transferred to the room temperature and a dark place for 48 h. For expression analysis, plants were grown first on Fahraeus medium [31] for one week and then transferred to vermiculite (Sludyanaya Fabrika, Saint-Petersburg, Russia)-containing pots (3–4 plants per a pot) which were also moistened with nitrogen free Fahraeus medium in the growth chambers under a 16 h photoperiod at 21 °C (75% relative humidity). Ten days after germination, each plant was inoculated with 1 mL of *Sinorhizobium meliloti* strain Sm2011 liquid culture (grown in YEM (Yeast Extract Mannitol) [32] medium up to OD600 = 0.7) was used. Non-inoculated control roots together with the inoculated roots were harvested and used for RNA extraction.

*Agrobacterium rhizogenes*-mediated plant transformation was carried out according to our previous description [33].

Nitrate treatment of *M. truncatula* plants was performed in a hydroponic system. First, plants were grown on plates containing Fahraeus medium [31] for one week and then transferred to the hydroponic system, containing nitrogen-free liquid Fahraeus medium. After 4 days, KNO_3_ was added to the medium to a final concentration of 10 mM. Plant roots were harvested in 24 h after KNO_3_ treatment for gene expression analysis, and untreated plants was used as a control.

### 4.2. RNA Extraction and cDNA Synthesis

RNA extraction was performed using an RNeasy Plant Mini Kit (Qiagen, Hilden, Germany) according to the manufacturer’s instructions. DNase treatment was conducted using Rapid Out DNA Removal Kit (Thermo Fisher Scientific, Waltham, MA, USA). The concentration and quality of extracted RNA was measured using a NanoDrop 2000c ultraviolet-visible (UV-Vis) Spectrophotometer (Thermo Scientific, Waltham, MA, USA) and equal amount of RNA was used for cDNA synthesis. cDNA synthesis was carried out using a Revert Aid Reverse Transcriptase kit (Thermo Fisher Scientific, Waltham, MA, USA).

### 4.3. Quantitative Reverse Transcription Polymerase Chain Reaction (qRT-PCR) Analysis

qRT-PCR analysis was performed using CFX-96 real-time PCR detection system with a C1000 thermal cycler (Bio-Rad Laboratories, Alfred Nobel Drive Hercules, CA, USA) with SYBR Green intercalating dyes (Sintol, Moscow, Russia). The data were analyzed by the CFX Manager software (Bio-Rad Laboratories, Alfred Nobel Drive Hercules, CA, USA) with the 2^−ΔΔ^ Ct method [34]. Actin (Medtr7g026230) and ubiquitin (Medtr4g091580) genes were used as reference genes. All the qRT-PCRs were undertaken in three technical repeats. Primers were designed using primer3 (http://bioinfo.ut.ee/primer3-0.4.0/) and Vector NTI Advance 10 program (InforMax, http://www.informaxinc.com, Thermo Fisher Scientific, Waltham, MA, USA), and were synthesized by Evrogen (http://www.evrogen.com, Moscow, Russia). Primers used for qRT-PCR are listed in Table S1. Dissociation curves (55–95 °C) were used to confirm the specificity of PCR amplification.

### 4.4. Molecular Cloning

The CDS sequence of *MtCLE35* (Medtr2g091125.1) was amplified using high fidelity Phusion polymerase (Thermo Fisher Scientific, Waltham, MA, USA) with adding attB site (underlined) to the forward and reverse primers (MtCLE35_CDS_FOR: 5’-AAAAAAGCAGGCTTCATGGCAAACACACAAATAACTATATTT-3’ and MtCLE35_CDS_REV: 5’-CAAGAAAGCTGGGTTCTACTTGTTTTGTGGACCTGCA-3’) and then cloned into the entry vector pDONR221 (Thermo Fisher Scientific, Waltham, MA, USA). Then the CDS was cloned from entry vector to the destination vector pB7WG2D (containing 35S promoter for overexpression and GFP cassette to select transgenic plants under fluorescence) using LR Clonase™ II enzyme (Thermo Fisher Scientific, Waltham, MA, USA) The construct was verified by sequencing. The resulting vector was transformed to *Agrobacterium rhizogenes* strain MSU440.

### 4.5. GFP (Green Fluorescent Protein) Fluorescence Detection

GFP detection and imaging was performed using Leica M205 FA fluorescence stereo microscope (www.leica-microsystems.com, Leica Microsystems, Wetzlar, Germany). The images were processed using the ImageJ program (National Institutes of Health, Bethesda, MD, USA) [35].

### 4.6. Statistical Methods and Computer Software

In the gene expression assay during nodulation at each time points four plants were used in each biological repeat (both for inoculated and non-inoculated plants). One-way analysis of variance (ANOVA) and Student’s *t*-test were used to compare gene expression levels. The box plot for nodule number in *MtCLE35*-overexpressing and control (GUS-overexpressing) plants was drawn in RStudio (https://rstudio.com/, Boston, MA, USA). A Mann–Whitney *U* test was used to compare nodule numbers in *MtCLE35*-overexpressing and control (GUS-overexpressing) plants; in each group from 15 to 25 plants have been analyzed in each biological repeat. At least three independent biological repeats were done for each experiment.

Multiple alignment of protein sequences was performed using UGENE software (http://ugene.net/ru/, Novosibirsk, Russia) [36] with Clustal W algorithm [37]. For phylogenetic analysis, protein sequences were retrieved from Phytozome v12.1 for *Medicago truncatula* and from Genbank National Center for Biotechnological Information (NCBI) database2 for *Lotus japonicus* and *Glycine max*. Sequences were aligned using the MEGAX program (https://www.megasoftware.net/) with the Clustal W algorithm, and the phylogenetic tree was generated using Maximum Likelihood method with 1000 bootstrap replicates.

## 5. Conclusions

The *MtCLE35* gene is induced by both rhizobia inoculation and nitrate treatment in *Medicago truncatula*. MtCLE35 systemically suppresses nodulation in an AON-dependent manner, mediating the nitrate-induced inhibition of nodulation in *M. truncatula*.

## Supplementary Materials

The following are available online at https://www.mdpi.com/2223-7747/9/11/1456/s1. Table S1: Primers used for the expression analysis; Figure S1: Phylogenetic tree based on the protein sequences of *CLE* genes of *Medicago truncatula, Lotus japonicus,* and *Glycine max*. The tree was generated using Maximum likelihood algorithm with 1000 bootstrap replicates; Figure S2: The expression levels of the *MtCLE35* gene at different days post inoculation (dpi) in comparison to the non-inoculated roots (NI). Results are mean ± SEM of three technical repeats of one biological repeat, representative for three independent experiments; Figure S3: The expression levels of the *MtCLE35* gene in nodules in comparison with the root according to transcriptomic data obtained by LCM (Laser Capture Microdissection)-RNA-seq for *M. truncatula* (https://iant.toulouse.inra.fr/symbimics, [27]) (A), *MtCLE35* expression at 0, 4, 10, 14 and 28 dpi according to theSmall Secreted Peptide Gene Expression Atlas (SSP-GEA) available in The *Medicago truncatula* Small Secreted Peptide Database (https://mtsspdb.noble.org [28] (B); Figure S4: Examples of nodulation phenotypes of composite wild-type plants containing both transgenic GFP-positive control (*β-glucuronidase* (GUS))-overexpressing) (A and B) and *MtCLE35*-overexpressing (C–F) roots and non-transgenic GFP-negative roots. White arrows indicate non-transgenic GFP-negative roots exhibiting faint autofluorescence. Red arrows point at nodules on GFP-positive transgenic roots, yellow arrows point at nodules on GFP-negative non-transgenic roots; Figure S5: Examples of nodulation phenotypes of composite *sunn-4* mutant plants containing both transgenic GFP-positive control (*GUS*-overexpressing) (A and B) and *MtCLE35*-overexpressing (C–F) roots and non-transgenic GFP-negative roots. White arrows indicate non-transgenic GFP-negative roots exhibiting faint autofluorescence. Red arrows point at nodules on GFP-positive transgenic roots, yellow arrows point at nodules on GFP-negative non-transgenic roots.
