# Supplementary material for: Nitrate-Induced CLE Peptide Systemically Inhibits Nodulation in Medicago truncatula
Source: Plants (Basel). 2020 Oct 28;9(11):1456. doi: 10.3390/plants9111456 (PMC7692534; doi:10.3390/plants9111456)
Supplement: Supplementary file 1 [file plants-09-01456-s001.pdf]

Table S1. Primer used for the expression analysis.

gene	Gene ID	Forward primer	Reverse primer
MtCLE 35	*Medtr2g091125*	5'- GCAAGCTCGTACTCTCCAAC C-3'	5'- TGTTGATTTGCATCCTCGTG-3'
MtCLE12	*Medtr4g079630*	5'- CAACGTCTCTTGCATGAGTTA ATGG-3'	5'- ACCTGGTGAAAGCCTATCTCCT G-3'
*MtCLE13**	Medtr4g079610	5'- CCGAAGCCTTCTACAGAAAC ACG-3'	5'- TCTTGGTGGTGATCTTCCATTAT GC-3'

*primer sequences for MtCLE13 forward and reverse primer were used according to [12].

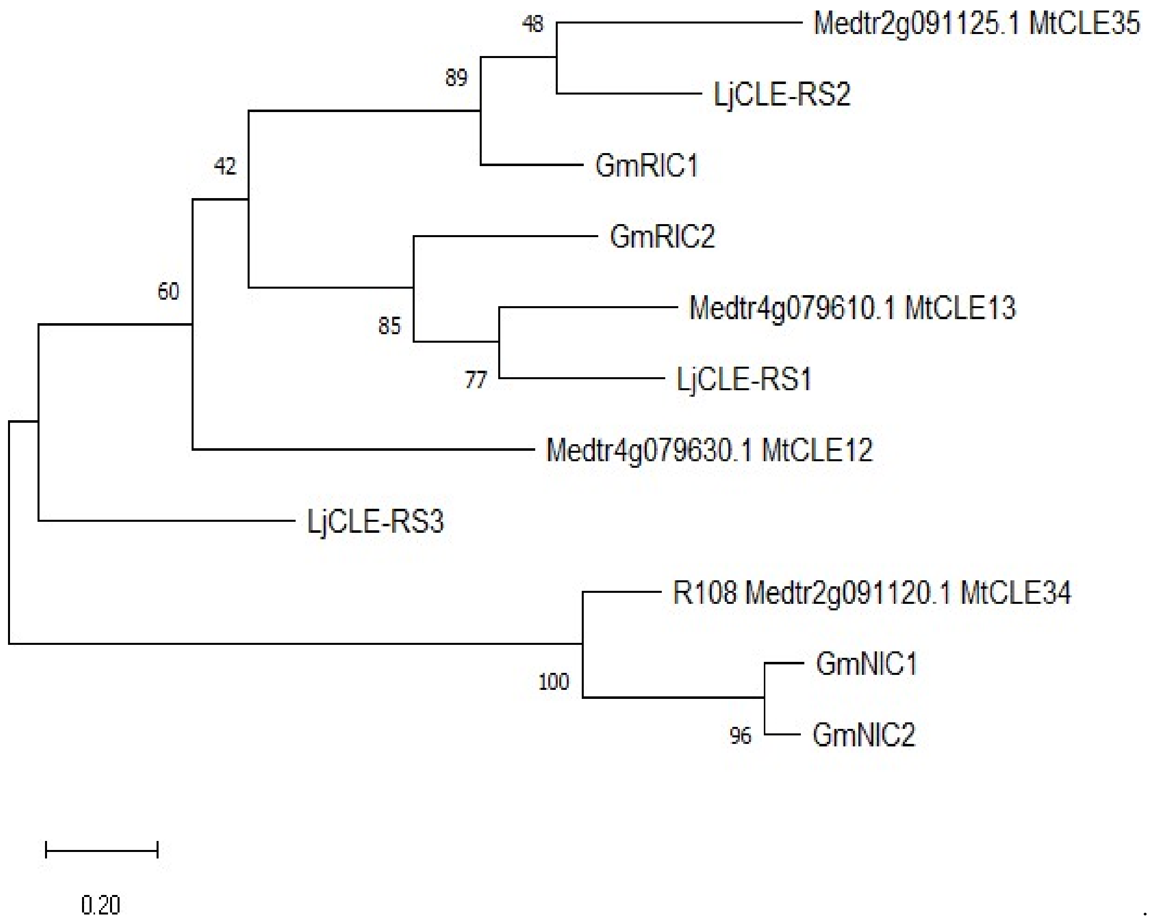

Figure S1. Phylogenetic tree based on the protein sequences of *CLE* genes of *Medicago truncatula, Lotus japonicus,* and *Glycine max*. The tree was generated using a maximum likelihood algorithm with 1000 bootstrap replicates.

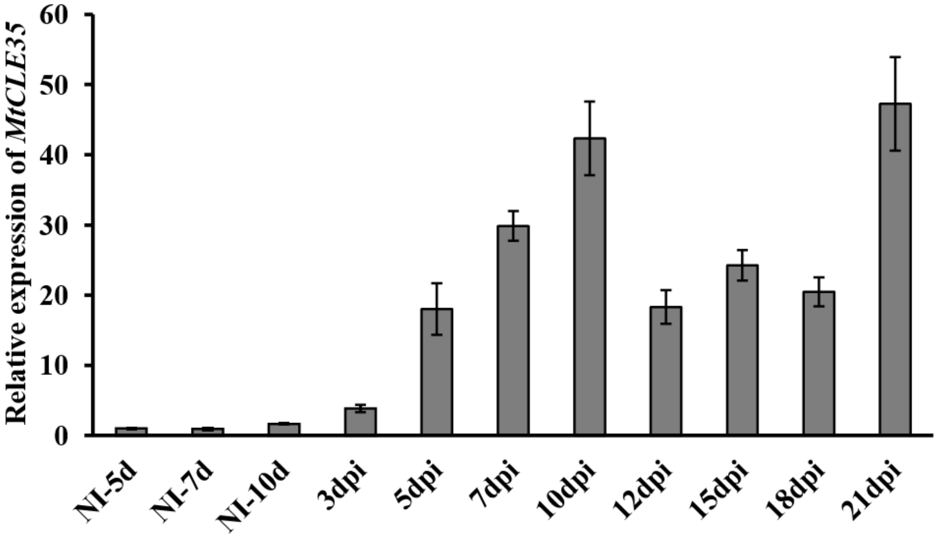

Figure S2. The expression levels of the *MtCLE35* gene at different days post inoculation (dpi) in comparison to the non-inoculated (NI) roots. Results are mean ± SEM of three technical repeats of one biological repeat, representative for three independent experiments.

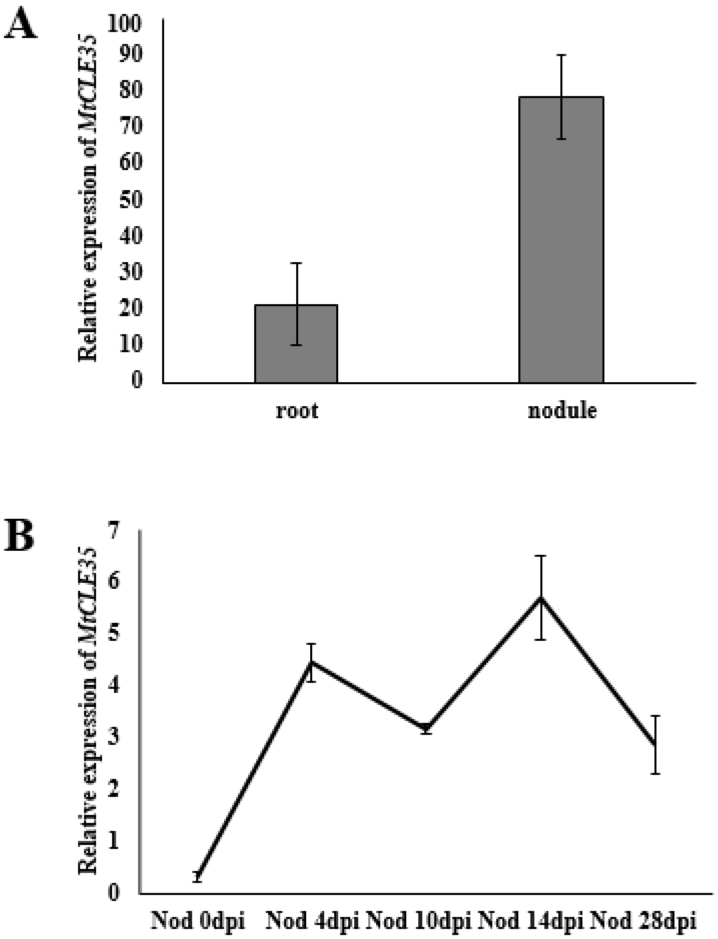

Figure S3. The expression levels of the *MtCLE35* gene in nodules in comparison with the root according to transcriptomic data obtained by LCM (Laser Capture Microdissection)-RNA-seq for *M. truncatula* (https://iant.toulouse.inra.fr/symbimics,[27]) (**A**), *MtCLE35* expression at 0, 4, 10, 14 and 28 dpi according to Small Secreted Peptide Gene Expression Atlas (SSP-GEA) available in The *Medicago truncatula* Small Secreted Peptide Database (https://mtsspdb.noble.org/, [28] (**B**).

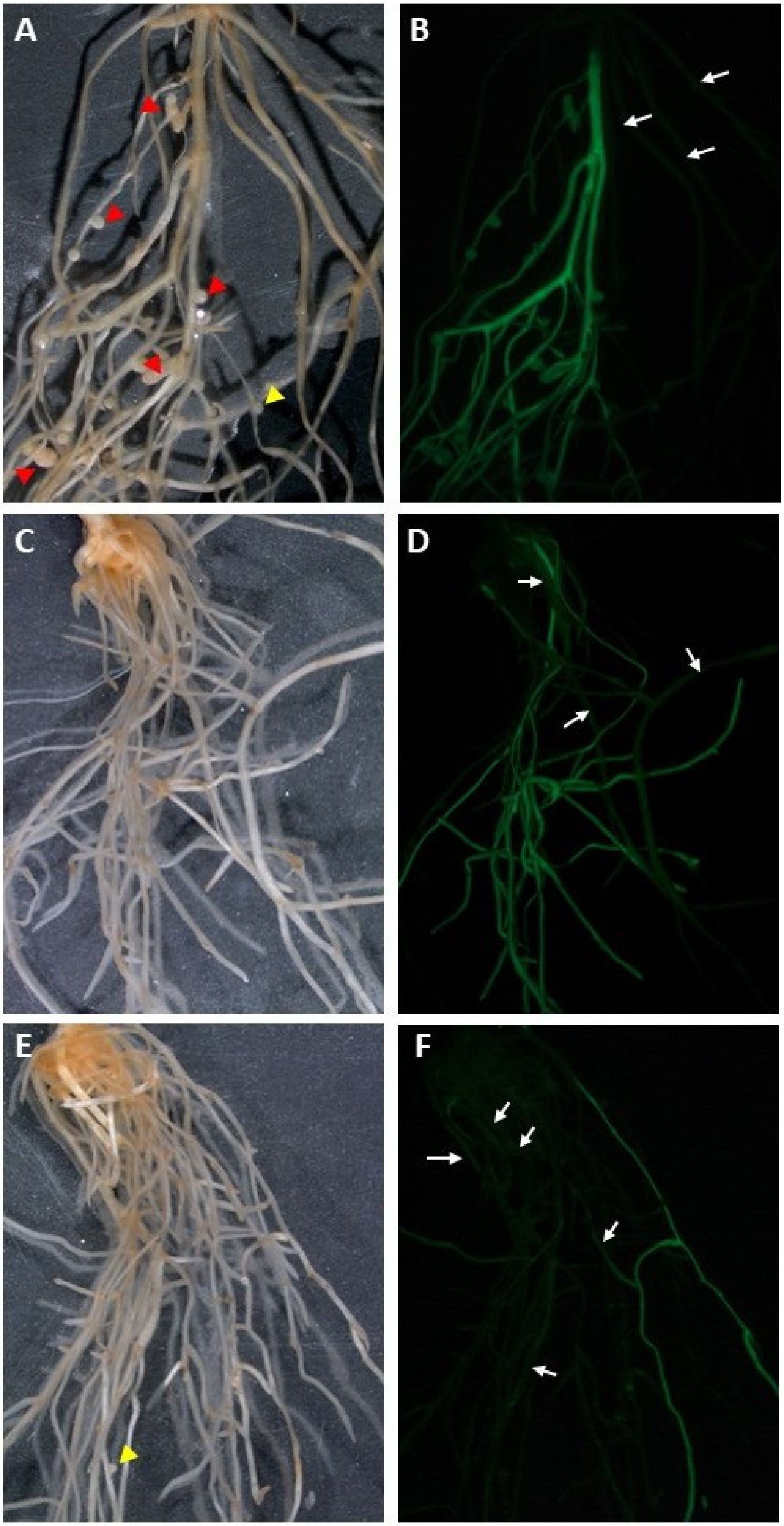

Figure S4. Examples of nodulation phenotypes of composite wild-type plants containing both transgenic GFP-positive control (*GUS* (*β-glucuronidase*)-overexpressing) (**A** and **B**) and *MtCLE35*- overexpressing (**C–F**) roots and non-transgenic GFP-negative roots. White arrows indicate non-transgenic GFP-negative roots exhibiting faint autofluorescence. Red arrows point at nodules on GFP-positive transgenic roots, yellow arrows point at nodules on GFP-negative non-transgenic roots.

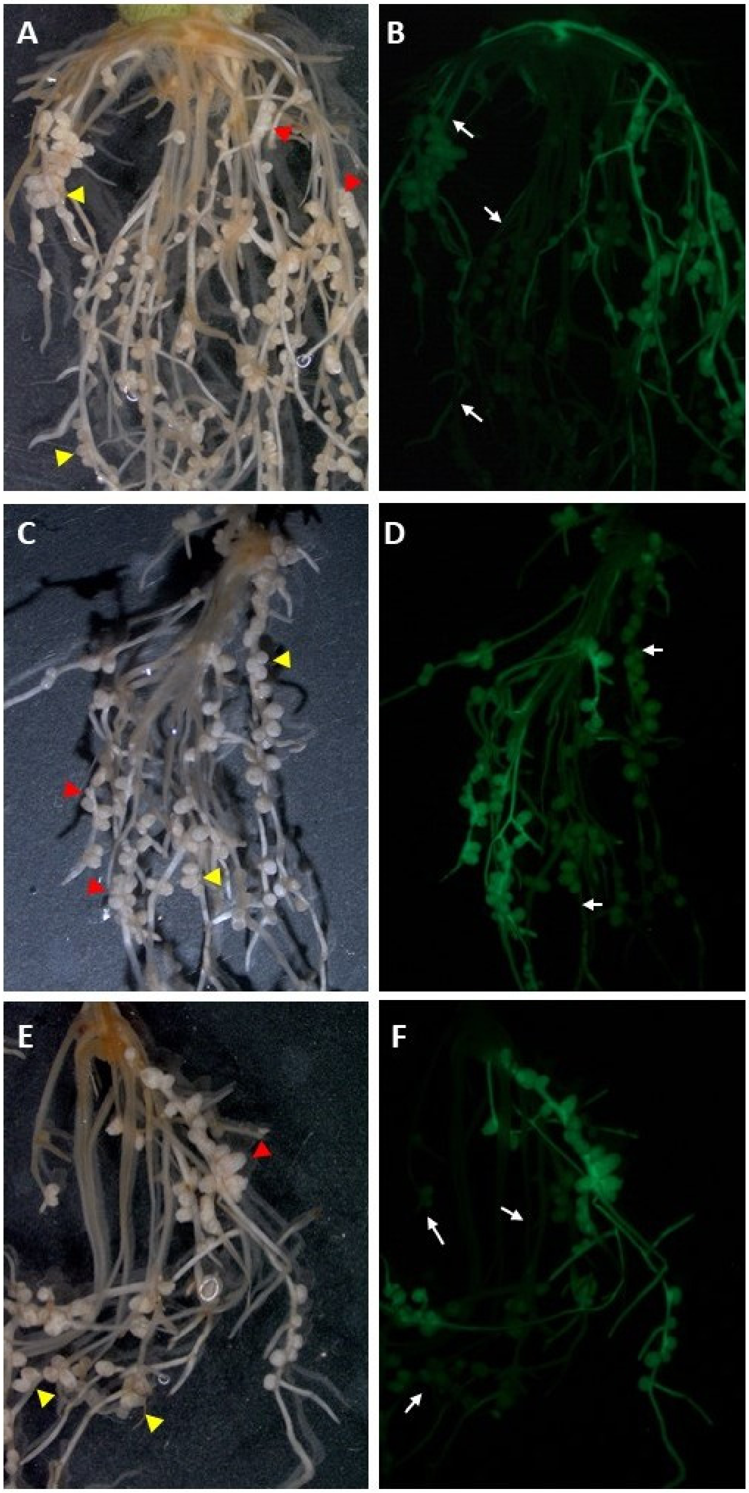

Figure S5. Examples of nodulation phenotypes of composite *sunn-4* mutant plants containing both transgenic GFP-positive control (*GUS*-overexpressing) (**A** and **B**) and *MtCLE35*-overexpressing (**C–F**) roots and non-transgenic GFP-negative roots. White arrows indicate non-transgenic GFP-negative roots exhibiting faint autofluorescence. Red arrows point at nodules on GFP-positive transgenic roots, yellow arrows point at nodules on GFP-negative non-transgenic roots.
